# Lessons Learned From COVID‐19: Never Lower the Guard in Promoting Hypertension Awareness

**DOI:** 10.1111/jch.70100

**Published:** 2025-07-20

**Authors:** Giacomo Pucci, Chiara Giacinti, Guido Grassi

**Affiliations:** ^1^ Unit of Internal and Translational Medicine Santa Maria Terni Hospital Terni Italy; ^2^ Department of Medicine and Surgery University of Perugia Perugia Italy; ^3^ Department of Medicine and Surgery University Milano‐Bicocca Milan Italy

1

“The race against a silent killer” is the sub‐title assigned to the 2023 World Health Organization Global Report on Hypertension, that synthetizes the complex challenges and interventions required to address the global burden of hypertension [1]. One of the most pressing challenges highlighted in this report is the ongoing need to raise awareness about hypertension, as a silent cardiovascular risk factor. Since more than one‐third of people with hypertension are unaware of their status, blood pressure (BP) screening is vital and much emphasis should be put on regular BP monitoring. Public screening initiatives and health education campaign are other key strategies for addressing this issue, both at the individual and at population level.

In 2019, before the COVID‐19 pandemic, a report from the Non‐Communicable Disease (NCD) Risk Factor Collaboration [2] revealed a substantial and rather stable increase in hypertension awareness over the last four decades. Notably, each improvement in awareness was associated with a corresponding decline in the proportion of individuals with undiagnosed or untreated hypertension. Reasons of this success were multiple: broader implementation over time of clinical guidelines featuring simplified clinical recommendations; the availability of newer fixed‐dose combination of antihypertensive drugs with improved efficacy, reduced treatment complexity and fewer side effects; the introduction of national programs focused on hypertension education and screening, as it was the case of the Canadian healthcare system [3]; and many other aspects.

During COVID‐19 pandemics, such scenario was suddenly and dramatically reversed. Policies designed to limit person‐to‐person contact significantly altered patient's health‐seeking behavior and reduced the frequency of in‐person visits to general practitioners [4]. Many patients forgot routine care because of concerns about COVID‐19 [5]. Physician‐driven factors were also involved, such as offices closures and reduced availabilities for appointments, which were only in part mitigated by delivery of telehealth [6]. Health literacy developments including educational health campaigns raising awareness against non‐communicable diseases, were often postponed [7], making way to informational campaigns focused on the risks associated with SARS‐CoV‐2 transmission. Finally, issues with the drug supply chain have been also reported during COVID‐19 [8].

As a result, rates of hypertension diagnosis and treatment initiation dramatically fell during the COVID‐19 pandemic, especially in the early phase [9] and BP control was reduced by on average 5%–7%, according to the results of a survey conducted by the BP Control Laboratory Surveillance System including 1.7 million patients from 24 US health system [10].

In the present issue of the Journal of Clinical Hypertension, Essa et al. contributed a key piece to this puzzle [11]. They analyzed data about hypertension prevalence, awareness and control in a sampling US population participating to the National Health and Nutrition Examination Survey (NHANES). The study included 14 449 participants representing 237.2 million US adults ≥18 years. The NHANES is considered one of the most comprehensive and reliable sources of health data in the United States, since it applies a complex, multistage probability sampling design that ensures data are representative of the US population.

By comparing results collected in the pre‐pandemic (2017–2020) to those in the post‐pandemic (2021–2023) period, the authors showed no changes in hypertension prevalence and a non‐significant decreasing trend in hypertension awareness (from 57.7% to 53.7%) which was mainly driven by a significant −22% decline in hypertension awareness in the age range between 18 and 39 years. The authors also found a significant decrease in BP control among men, which they attributed to low awareness, although a formal interaction term analysis was not carried out.

Hypertension awareness is particularly challenging among young adults, especially among young men. In the NCD report, men aged 40–49 years showed the lowest rate of hypertension awareness [2]. Among factors contributing to this poor result are a low perception of risk and a weak connection to the healthcare system, both associated with the misconception that hypertension can start early in life and, since it does not cause symptoms, is harmless. Another important factor is medical inertia, since young men are less likely to have their hypertension diagnosed by their physician, and less likely to receive information about lifestyle changes [12].

The opportunity to reflect on the findings provided by the authors, should reinforce our understanding that increasing hypertension awareness is essential to reduce the global burden of hypertension and should be pursued every day. However, it remains one of the most difficult goal to achieve, as it depends on complex, coordinated efforts involving all the interconnected stakeholders within the healthcare system. No one should ever lower their guard down, even briefly.
